# Effects of Nasal or Pulmonary Delivered Treatments with an Adenovirus Vectored Interferon (mDEF201) on Respiratory and Systemic Infections in Mice Caused by Cowpox and Vaccinia Viruses

**DOI:** 10.1371/journal.pone.0068685

**Published:** 2013-07-09

**Authors:** Donald F. Smee, Min-Hui Wong, Brett L. Hurst, Jane Ennis, Jeffrey D. Turner

**Affiliations:** 1 Department of Animal, Dairy and Veterinary Sciences, Institute for Antiviral Research, Utah State University, Logan, Utah, United States of America; 2 Defyrus, Inc., Toronto, Ontario, Canada; Public Health Agency of Canada, Canada

## Abstract

An adenovirus 5 vector encoding for mouse interferon alpha, subtype 5 (mDEF201) was evaluated for efficacy against lethal cowpox (Brighton strain) and vaccinia (WR strain) virus respiratory and systemic infections in mice. Two routes of mDEF201 administration were used, nasal sinus (5-µl) and pulmonary (50-µl), to compare differences in efficacy, since the preferred treatment of humans would be in a relatively small volume delivered intranasally. Lower respiratory infections (LRI), upper respiratory infections (URI), and systemic infections were induced by 50-µl intranasal, 10-µl intranasal, and 100-µl intraperitoneal virus challenges, respectively. mDEF201 treatments were given prophylactically either 24 h (short term) or 56d (long-term) prior to virus challenge. Single nasal sinus treatments of 10^6^ and 10^7^ PFU/mouse of mDEF201 protected all mice from vaccinia-induced LRI mortality (comparable to published studies with pulmonary delivered mDEF201). Systemic vaccinia infections responded significantly better to nasal sinus delivered mDEF201 than to pulmonary treatments. Cowpox LRI infections responded to 10^7^ mDEF201 treatments, but a 10^6^ dose was only weakly protective. Cowpox URI infections were equally treatable by nasal sinus and pulmonary delivered mDEF201 at 10^7^ PFU/mouse. Dose-responsive prophylaxis with mDEF201, given one time only 56 d prior to initiating a vaccinia virus LRI infection, was 100% protective from 10^5^ to 10^7^ PFU/mouse. Improvements in lung hemorrhage score and lung weight were evident, as were decreases in liver, lung, and spleen virus titers. Thus, mDEF201 was able to treat different vaccinia and cowpox virus infections using both nasal sinus and pulmonary treatment regimens, supporting its development for humans.

## Introduction

In a previous publication, we reported that an adenovirus vectored mouse interferon (mDEF201), administered intranasally (i.n.), was capable of protecting mice infected i.n. with vaccinia (WR strain) virus [1]. This infection model used a 50-µl virus inoculum that induced primarily a lower respiratory infection (LRI). There was also a less-severe upper respiratory infection (URI), since the i.n.-administered liquid must pass through the snout on its way to the lungs. mDEF201 doses of 10^5^ to 10^7^ PFU/mouse administered to the lungs in a 50-µl volume 24 h prior to virus challenge protected 90–100% of mice from death. The same report described giving a single 10^7^ dose of mDEF201 56 days (8 weeks) prior to infection, with a 90% survival outcome.

The results of the prior studies left a number of unanswered questions about the utility and benefits of mDEF201 in mice that might relate to the treatment of humans with DEF201 (the human interferon form of the vector). The first question pertains to the treatment volume of mDEF201 that was given to mice. A 50-µl treatment volume in a mouse that has been shown to deliver much of the dose to the lungs [2], if scaled up to a human based on body weight would be excessive. The preferred treatment volume for humans should be small so as to be comfortable to receive, and delivered via an intranasal delivery device that delivers to the nasal mucosa. The second question relates to the types of infections that are treatable with mDEF201. We primarily looked at one virus model, a vaccinia virus LRI induced by i.n. virus inoculation [1]. We have developed other infection models with cowpox and vaccinia viruses in healthy immunocompetent mice. Systemic infections were produced by intraperitoneal infection with virus [3], and primarily LRI and URI infections were developed by using either a large (50-µl) or small (5-µl) i.n. infection volume [4]. These models are useful to address the efficacy of mDEF201 in treating various types of infections caused by cowpox and vaccinia viruses, as well as to determine whether changes in mDEF201 delivery nasal sinus versus lung affected the efficacy of the drug in protecting against these challenge agents.

A final question that was raised by the previous studies [1] pertains to the long acting prophylactic effect of mDEF201, where a single 10^7^ PFU/mouse dose given 56 days prior to vaccinia virus challenge provided remarkable protection. Lower doses of mDEF201 have not been assessed in this model, and as such needed to be tested to determine the extended protection of the vector if given 8 weeks before infection.

As indicated previously [1], a needle-free, single dose drug capable of achieving steady-state method of delivering interferon would maximize the therapeutic benefits of IFN, while minimizing the bolus-induced toxicity. Additionally, i.n. delivery of the adenovirus vector bypasses pre-existing immunity to the vector [5]. mDEF201 has already shown efficacy in mice infected with other viruses such as Ebola [6], Western equine encephalitis virus [7], Venezuelan equine encephalitis virus [8], and SARS [9]. The human form, DEF201, which is also active in hamsters, has been used to treat yellow fever virus infections [10], arenavirus infections [11], and bunyavirus infections [12]. Guinea pigs infected with Ebola virus were successfully treated with a combination of vaccine plus DEF201 [6].

Although there are antiviral drugs under development for orthopoxvirus infections (variola and monkeypox, bioterror agents) [13], human DEF201 appears to hold promise as both a prophylactic and early treatment agent. The experiments that are presented here further elucidate the ability of mDEF201, delivered to the nasal sinus or lung to combat different forms of poxvirus infections.

## Materials and Methods

### Ethics Statement

The experiments were conducted in accordance with Protocol 552 approved by the Institutional Animal Care and Use Committee of Utah State University. The work was performed in the Biosafety Level 2 area of the AAALAC-accredited Laboratory Animal Research Center of Utah State University in accordance with the National Institutes of Health Guide for the Care and Use of Laboratory Animals [14]. All individuals involved in working with infected animals and cells received prior vaccination with the standard smallpox vaccine.

### Animals

Female BALB/c mice (Charles River Labs, Wilmington, MA) weighing approximately 13–15 g at the time of first treatment were used for most studies. Mice receiving extended prophylaxis for 8 weeks prior to infection weighed 19–20 g at that time. They required a slightly higher virus challenge dose for lethality, as indicated below. After a 48-hour quarantine period after initial receipt, the animals were randomly assigned to cages.

### Test Materials

The adenovirus vectored mouse interferon alpha, mDEF201, was prepared at the Robert Fitzhenry Vector Laboratory (McMaster University, Hamilton, Canada). The methods of preparation have been described previously [7]. Briefly, the mouse interferon alpha gene (subtype 5) was cloned into a replication deficient Ad-5 vector (deletions of E1 and E3 genes), amplified in 293 cells and purified by cesium chloride gradient centrifugation. Stock solutions of mDEF201 were stored at −80°C and were thawed on ice and diluted in saline to the appropriate dose immediately prior to a single administration. Gilead Sciences (Foster City, CA) provided the positive control compound cidofovir.

### Virus

Vaccinia virus (WR strain) was purchased from the American Type Culture Collection (ATCC, Manassas, VA). Cowpox virus (Brighton strain) was obtained from John Huggins (Ft. Detrick, Frederick, MD). The cowpox virus contained a syncytium forming (SF) variant that was isolated as described previously [15], and was used for these studies. The viruses were propagated in African green monkey kidney (MA-104) cells (from ATCC).

### Experimental Design for Animal Studies

Mice were anesthetized with ketamine/xylazine (50/5 mg/kg) by intraperitoneal (i.p.) injection for i.n. treatments and i.n. infection. I.p. infections required no anesthesia. Amounts of infecting virus (PFU/mouse) to achieve complete lethality varied with virus type, infection route, and infection volume, as follows: vaccinia (100-µl i.p.) - 5×10^6^, vaccinia (50-µl i.n.) –1×10^5^, cowpox (10-µl i.n.) –1×10^6^, and cowpox (50-µl i.n.)- 5×10^5^. A long-term (8-week) prophylaxis study that was performed used aged mice, which required an i.n. vaccinia virus challenge dose of 2.5×10^5^ PFU/mouse to cause complete mortality. mDEF201 was administered i.n. to the nasal sinus or lung 24 h prior to vaccinia virus exposure for short-term prophylaxis, or 56 days prior to infection for long term prophylaxis. Placebo-treated mice were given saline by i.n. route. All animals were weighed every other day starting the day before and during the 21-day infection period. There were 10 mice in each mDEF201 or cidofovir treated group and 20 placebos per experiment.

In the long-term prophylaxis study, separate animals (5 mice per group) were maintained for determination of tissue virus titers, lung hemorrhage scores, and lung weights. These mice were sacrificed for removal of tissues after 5 days of infection. Lungs were given a hemorrhage score (color change from pink to plum which occurs regionally in the lung rather than gradually over the entire lung) ranging from 0 (normal) to 4 (entire lung affected). Lungs, spleens, livers, and snouts were weighed prior to homogenization that releases infectious virus for titration. Homogenization of soft tissues was performed in 1 ml of cell culture medium using a stomacher. Snouts were ground in 1 ml of medium using sterilized mortars and pestles. Samples were serially diluted in 10-fold increments and plaque titrated in 12-well microplates of Vero cells. Plaques were stained at three days with 0.2% crystal violet in 10% buffered formalin, followed by counting the plaques with the aid of a light box. Plaque numbers were converted to PFU per gram of infected tissue.

### Statistical Analysis

Initially, survivor numbers were compared by the Mantel-Cox log-rank test. When statistical significance was found, pairwise analyses were performed using the Gehan-Breslow-Wilcoxon test. Differences in tissue virus titers and lung weights were statistically analyzed by the Tukey-Kramer multiple comparisons test. Lung hemorrhage scores were analyzed by the two-tailed Mann-Whitney U-test. Analyses were performed using the InStat® and Prism 5.0® computer software programs (GraphPad Software, San Diego, CA), comparing treated and placebo groups.

## Results

### Effects of mDEF201 Prophylaxis Delivered via Nasal Sinus or Lung on a Vaccinia Virus LRI

**Figure 1:**
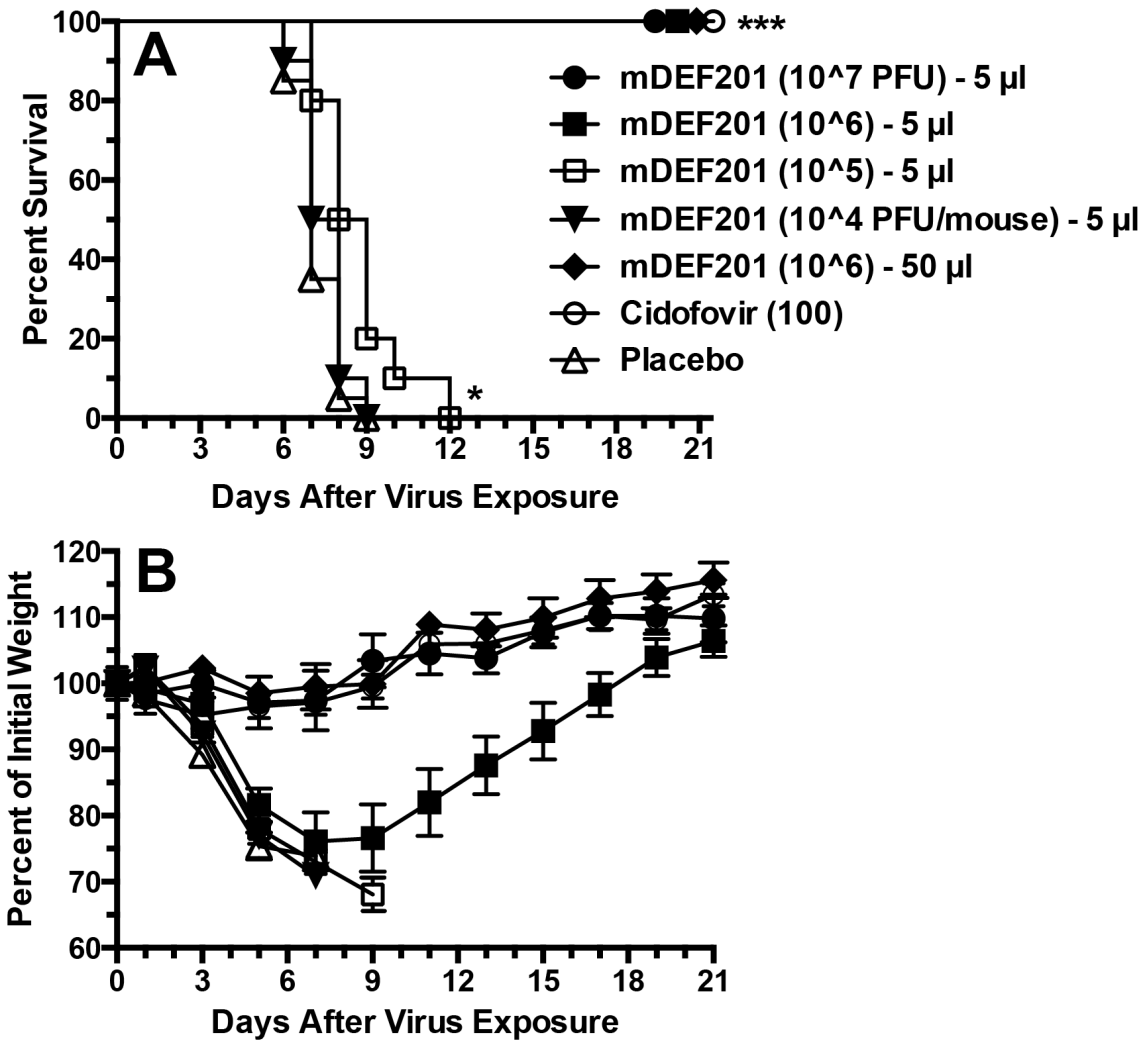
Effects of treatment with mDEF201 (5- and 50-µl) and cidofovir on survival (A) and body weight change (B) during a vaccinia virus lower respiratory infection (initiated by a 50-µl i.n. virus inoculation) in mice. Intranasal treatments with mDEF201 (PFU/mouse) were given one time only 24 hours prior to virus exposure. Cidofovir (mg/kg/day) was administered i.p. once a day for two days at 4 and 24 hours after infection. Figure 1B shows mean values ± SEM. There were 10 treated animals per group and 20 placebos at the start of the experiment. *P<0.05, ***P<0.001, compared to placebo.

Previously we reported that high (50-µl) volume mDEF201 treatments were effective against vaccinia virus infections initiated by 50-µl virus infection volumes [1]. This virus infection method causes more of a lung infection (LRI) that a sinus infection (URI), and virus dissemination to other tissues (such as liver and spleen) is lower as well. In Figure 1 a 5-µl nasal sinus treatment volume was tested for efficacy, since this volume would be more representative if scaled up to a human dose. For comparative purposes, one dose of mDEF201 delivered to the lungs in a 50-µl volume and i.p. cidofovir were also evaluated. The two higher doses of mDEF201 protected all mice in the groups from death, as did cidofovir (Figure 1A). At 10^6^ PFU/mouse delivered either via nasal sinus or lung mDEF201 prevented mortality. However, severe weight loss was encountered in animals treated with the nasal sinus delivered drug (Figure 1B) but the weight was re-gained by 21 days. Delivered via the lung at 10^6^ PFU/mouse, mDEF201 protected against weight loss as well as a nasal delivered 10^7^ dose and cidofovir.

### Effects mDEF201 Prophylaxis Delivered via Nasal Sinus or Lung on Vaccinia Virus Systemic Infections

**Figure 2:**
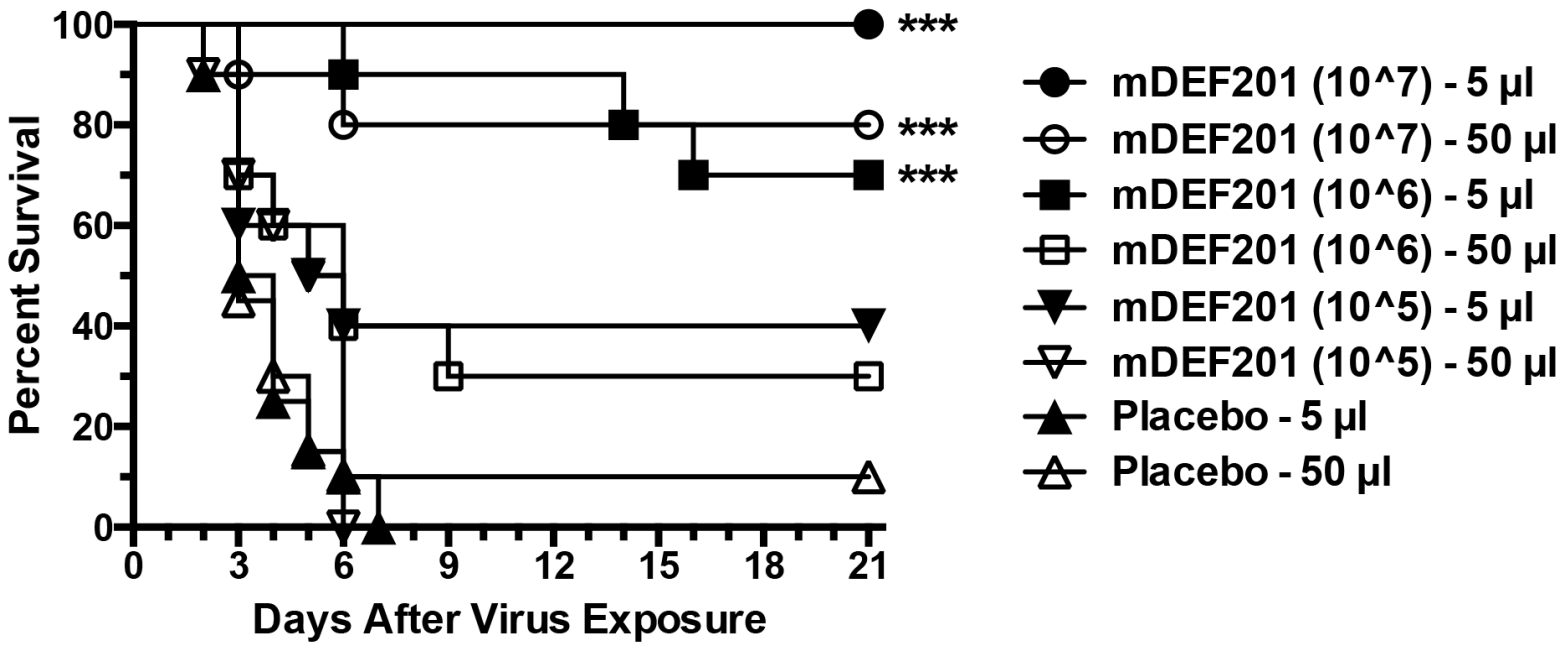
Effects of treatment with mDEF201 on survival from a vaccinia virus systemic infection (initiated by a 100-µl i.p. virus inoculation) in mice. Intranasal 5- and 50-µl treatments with mDEF201 (PFU/mouse) were given one time only 24 hours prior to virus exposure. There were 10 treated animals per group and 20 placebos per group at the start of the experiment. ***P<0.001, compared to placebo.

Vaccinia virus systemic infections that were initiated by i.p. virus challenges can be effectively managed prophylactically with mDEF201. Liver, lungs, and spleen are major targets of this infection [3]. mDEF201 deposited primarily either in the sinuses (5-µl volume) or in the lungs (50-µl volume) would have to generate interferon that ultimately needs to reach the liver and other organs for protection. Complete protection was afforded by the 10^7^ dose of mDEF201 administered to the nasal sinus (Figure 2) while lower doses provided partial protection. Each dose of mDEF201 delivered to the lungs was less protective (11/30 survived) than the same dose delivered to the nasal sinus (21/30 survived) (a difference of P<0.02 by two-tailed Fisher’s exact test). Thus, the nasal sinus delivered mDEF201 was more effective in treating this systemic infection, suggesting that more interferon was produced by the mDEF201 expression or that the gene product, interferon was better distributed when produced in the nose.

### Effects of mDEF201 Prophylaxis on a Cowpox Virus LRI

**Figure 3:**
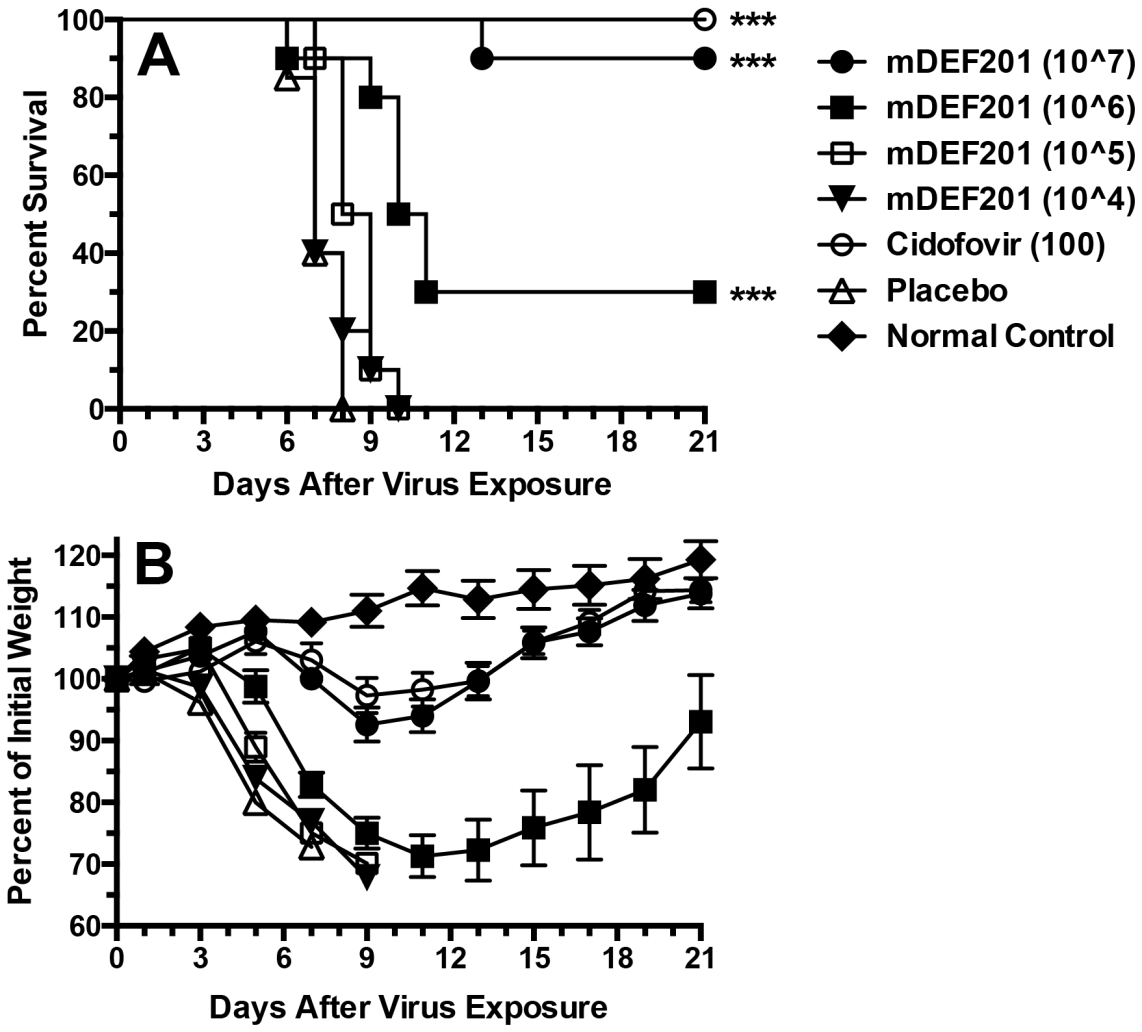
Effects of treatment with mDEF201 on survival (A) and body weight change (B) during a cowpox virus lower respiratory infection (initiated by a 50-µl i.n. virus inoculation) in mice. Intranasal (50-µl) treatments with mDEF201 (PFU/mouse) were given one time only 24 hours prior to virus exposure. Cidofovir (mg/kg/day) was administered once a day for 2 days starting 4 hours after virus challenge. Figure 3B shows mean values ± SEM. There were 10 treated animals per group, 20 placebos, and 6 normal control mice (uninfected, untreated) at the start of the experiment. ***P<0.001, compared to placebo.

Up to this point in our research in the orthopoxvirus family we had only used mDEF201 to treat a vaccinia virus infection in mice. Since cowpox and vaccinia viruses are related and vaccinia was treatable with mDEF201, it could be assumed that mDEF201 should also be effective against the cowpox virus infection. To investigate this, mDEF201 was administered to the lung before cowpox virus infection, similar to what was reported for vaccinia virus infections [1] (see also Figure 1). A high degree of protection was seen with the 10^7^ PFU dose, and much less with the 10^6^ PFU dose (Figure 3A). Cidofovir was completely protective. Slight body weight loss during infection was seen with the 10^7^ dose of mDEF201 and cidofovir (Figure 3B). Lower doses of mDEF201 could not prevent severe infection-induced weight loss. A comparison of the degree of protection afforded by pulmonary delivered 10^6^ dose of mDEF201 against vaccinia (Figure 1) and cowpox (Figure 3) viruses suggests that cowpox virus infection was more difficult to treat.

### Effects of mDEF201 Prophylaxis on Cowpox Virus URIs

**Figure 4:**
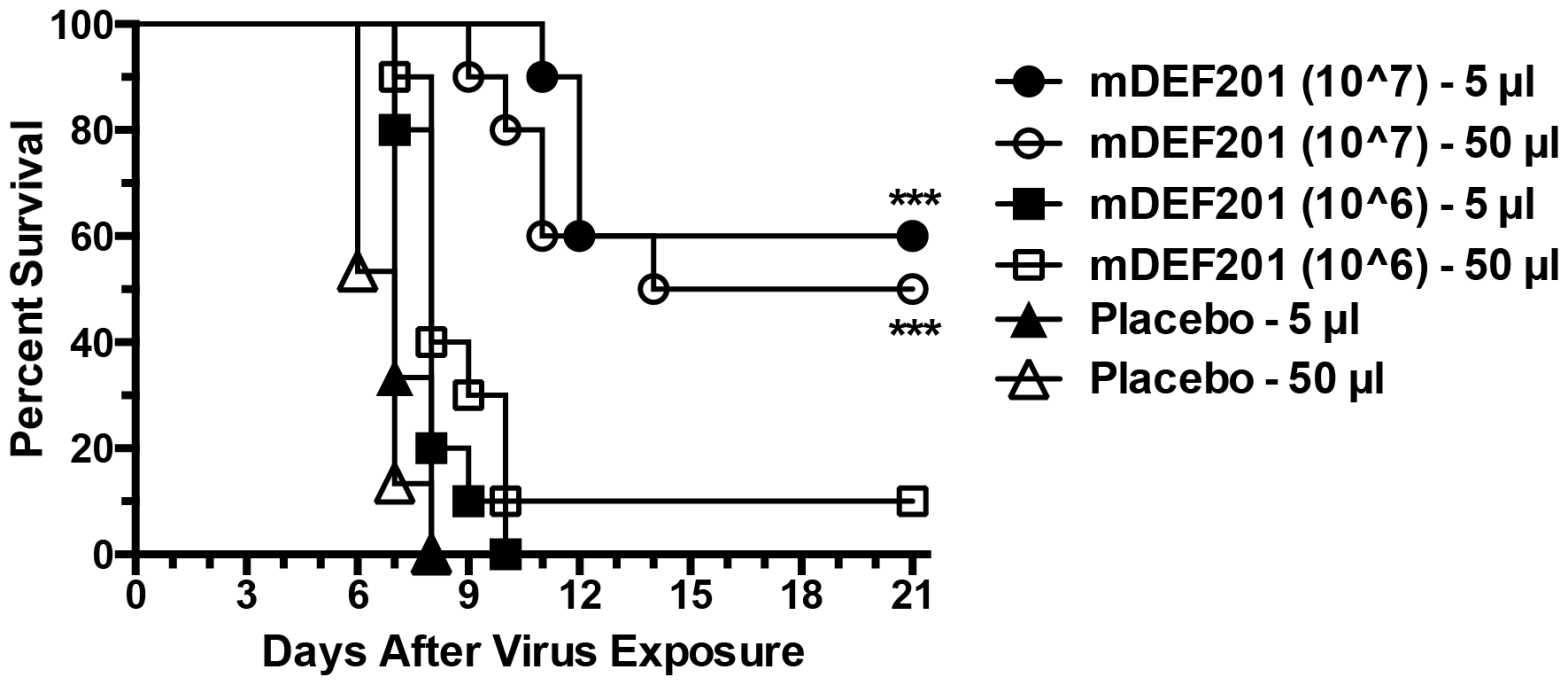
Effects of treatment with mDEF201 on survival from a cowpox virus upper respiratory infection (initiated by a 10-µl i.n. virus inoculation) in mice. Intranasal (5- and 50-µl) treatments with mDEF201 were given one time only 24 hours prior to virus exposure. There were 10 treated animals per group and 20 placebos per group at the start of the experiment. ***P<0.001, compared to placebo.

We found that the syncytium forming strain of cowpox virus in a low volume inoculum works well for causing a lethal upper respiratory tract infection in mice [4], and there is minimal LRI involvement. In this report, URIs were treated with mDEF201 delivered to the nasal sinus (5 µl) or lung (50 µl). The small volume would concentrate the vector at the site of infection, whereas the large volume would deposit most of the vector more diffusely in the lungs. Once in the lungs, the interferon that was produced would have to circulate to the upper respiratory tract in order to exert a beneficial effect. Because mDEF201 has to pass through the sinuses en route to the lungs, there would be some of the vector deposited in the sinuses, but in a much lower amount and concentration. The results of this infection were enlightening. A dose of 10^7^ PFU/mouse provided only a partial protection from death (Figure 4), and the 10^6^ dose was ineffective. Nearly equivalent protection was afforded when a dose of 10^7^ PFU mDEF201 were administered via nasal sinus or lung. The results suggest that upper respiratory tract infections are more difficult to treat with mDEF201, even when very concentrated amounts of mDEF201 are administered locally.

### Long-term Prophylactic Effects of Different Doses of mDEF201 on a Vaccinia Virus LRI

**Figure 5:**
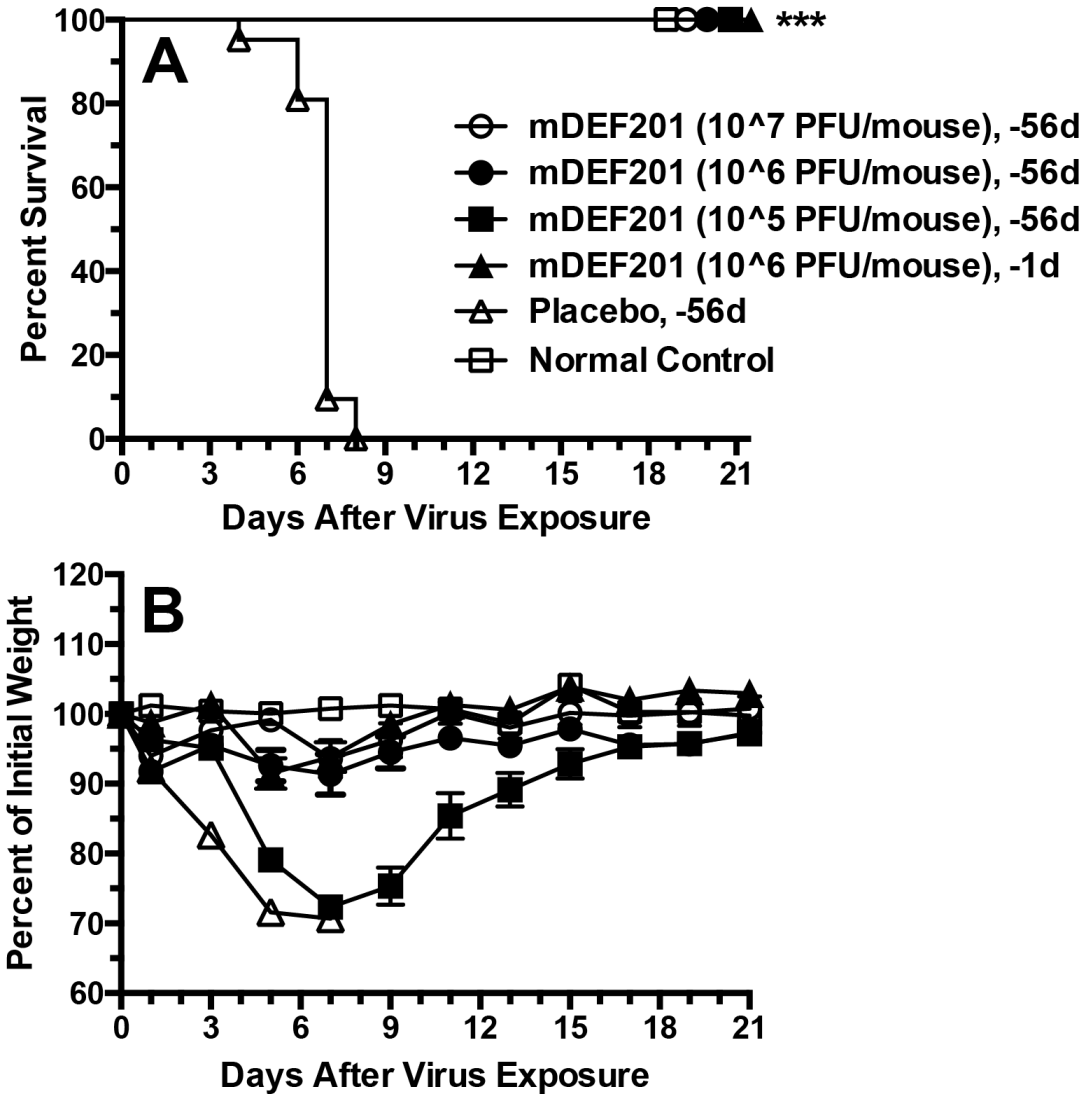
Effects of extended prophylaxis with different doses of mDEF201 on survival (A) and body weight change (B) during a vaccinia virus lower respiratory infection (initiated by a 50-µl i.n. virus inoculation) in mice. Single 50-µl i.n. treatments (PFU/mouse) were given on the day indicated prior to virus challenge. Figure 5B shows mean values ± SEM. There were 10 treated animals per group, 20 placebos, and 6 normal control mice (uninfected, untreated) at the start of the experiment. ***P<0.001, compared to placebo.

**Figure 6:**
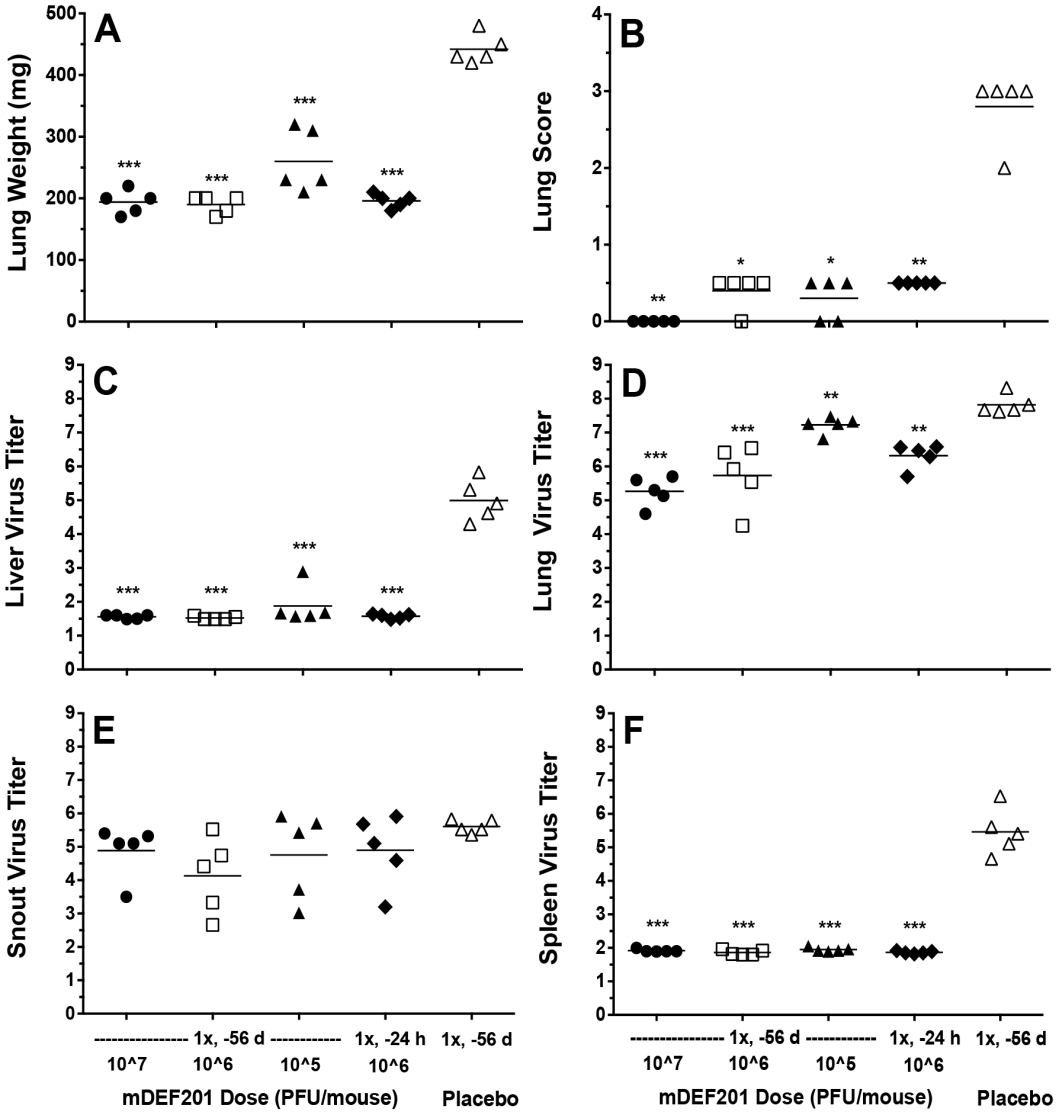
Effects of extended prophylaxis with different doses of mDEF201 on tissue parameters during a vaccinia virus lower respiratory infection (initiated by a 50-µl i.n. virus inoculation) in mice. Single 50-µl i.n. treatments (PFU/mouse) were given on the day indicated prior to virus challenge. Determinations were made five days after infection. A = lung weight, B = lung hemorrhage score, C = liver virus titer, D = lung virus titer, E = snout virus titer, F = spleen virus titer. Virus titers are expressed as log_10_ PFU/g of tissue. The limits of detection for the assays approached 10^2^ PFU/g (most liver and spleen samples from mDEF201-treated mice did not have detectable virus present). The horizontal bars on the figures represent means values (N = 5 per group). *P<0.05, **P<0.01, ***P<0.001, compared to placebo.

Previously we demonstrated that a single high (10^7^ PFU/mouse) dose of mDEF201 administered 8 weeks prior to virus exposure was highly protective against vaccinia virus infection in mice [1]. Here we determined the effects of lower doses on the same extended prophylactic window. mDEF201 was administered 56 days prior to virus challenge in three different doses, and at one effective dose one day prior to infection for comparison (Figure 5A). Surprisingly, even the 10^5^ dose of mDEF201 given 56 days pre-infection protected all animals from death. However, severe weight loss was seen in that group, whereas the higher dosage groups were largely protected from weight loss due to infection (Figure 5B). Other parameters were measured on day 5 post-infection in the same experiment (Figure 6). Lung weights and lung hemorrhage scores were significantly less in mDEF201-treated animals compared to placebo (Figures 6A and 6B, respectively). Liver virus titers were nearly completely suppressed, except for one liver from an animal treated with 10^5^ units of mDEF201 (Figure 6C). The highest levels of virus were detected in lungs of animals (Figure 6D), and treatment with all doses of mDEF201 reduced those levels significantly compared to placebo. The 10^5^ dose given 56 days pre-infection was least effective, as might be expected from the body weight loss data (Figure 5B). The 10^6^ dose administered 56 days pre-infection showed comparable virus titer reduction to the 10^6^ dose given 1 day prior to infection, which is remarkable. By multiple statistical comparisons, the 10^7^ dose of mDEF201 administered 56 days pre-infection was significantly different (P<0.05 or less) than all other treatments. Snout virus titers were not significantly reduced by mDEF201 treatments (Figure 6E), which may explain the incomplete efficacy achieved against upper respiratory tract infections (Figure 4). Finally, spleen virus titers were undetectable in animals treated with mDEF201 either 56 days or 1 day before virus challenge (Figure 6F).

## Discussion

In these studies, a single dose of mDEF201 was administered to mice via 5 µl to the nasal sinus or 50-µl to the lungs to combat different manifestations of cowpox and vaccinia virus infections, including primarily upper respiratory, primarily lower respiratory, and systemic infections. The purpose of evaluating nasal sinus delivery of mDEF201 in these experiments was to better model the preferred method of treatment of human infections with DEF201. The 50-µl volume in a mouse would be overwhelming when scaled up to an amount appropriate for humans. Thus, we wanted to determine whether the positive results with mDEF201 reported in our first publication [1] were influenced by the high treatment volume. What was found in the present set of experiments was that mDEF201 was very effective when administered in a low volume to the nasal sinus. The potency of the vector when delivered in a low volume relative to the high volume depended on the virus infection model. Against the vaccinia virus LRI, mDEF201 delivered to the nasal sinus required a 10-fold higher dose than that delivered to the lungs in order to protect equally against body weight loss during the infection (Figure 1B). Conversely, in the vaccinia virus systemic infection, mDEF201 delivered via nasal sinus were clearly superior to lung dosing (Figure 2). Against a cowpox virus URI, the different routes of administration appeared to be equivalent in efficacy (Figure 4).

This is the first report where mDEF201 is reported to be active against cowpox virus infections in mice. A comparison of the results of treatment of this virus with that of vaccinia virus treatment suggests that the cowpox virus infection is more difficult to treat, requiring higher doses of mDEF201 for efficacy. We also observed that the cowpox URI was only partially impacted by the highest dose of mDEF201 (Figure 4), regardless of whether the compound was administered in a concentrated form (5-µl) targeting the upper respiratory tract or a diluted form (50-µl) targeting the lower respiratory tract. Because equivalent protection was seen by both nasal sinus and pulmonary treatments, this indicates that the amount of interferon at the site of infection may not be the important factor, but rather how well the sinuses can mount an antiviral response. Although not precisely relevant to cowpox but instructive is the fact that vaccinia viral titers in the snouts of mice were not diminished by mDEF201 treatment (Figure 6E), suggesting that the efficacy of interferon in the sinuses is lower than in other tissues (e.g., in livers and spleens, see Figures 6C and 6F).

This report also provides additional enlightenment to the observations made previously about the remarkably long prophylactic effect of mDEF201 against vaccinia virus infections [1]. Earlier it was shown that a high (10^7^) dose of vector administered 56 days (8 weeks) prior to infection afforded a high degree of protection from death. We evaluated lower (10^5^ and 10^6^) doses of mDEF201 and found them to protect 100% of animals from death, although the lower dose could not protect against excessive weight loss (Figure 5B). Also demonstrated was the fact that the 10^6^ dose administered 8 weeks pre-infection provided a similar degree of viral titer reduction capacity as the same dose administered 1 day before virus exposure (Figure 6). Additionally, lung weights and lung hemorrhage scores in these mice were similarly improved (Figure 6). All of this points to the remarkably long acting effect of mDEF201 against vaccinia virus infections in mice. We have not performed similar cowpox virus infections with extensive pre-treatment to know if the compound is similarly effective. It is reported that other animals cannot be prophylactically protected nearly so far from the day of infection with unrelated viruses, such as SARS coronavirus-infected mice [9] and yellow fever virus-infected hamsters [16]. It should be pointed out that this extended prophylaxis appears to exist in the absence of measurable serum interferon levels [7]. We know that the adenovirus vector is still present in the snouts of mDEF201 treated mice 60 days after exposure (unpublished). Interferon, if present at very low levels, may be activating an immunological cascade that protects the animal from infection by inducing a long lasting antiviral state. Over 40 antiviral genes in multiple pathways have now been identified in cells that can play a virus-inhibitory role, depending upon the virus [17]. We plan to measure the genes that are stimulated by DEF201 or mDEF201 in our safety studies, and look for correlates to antiviral protection.

This set of experiments provides further evidence of the utility of mDEF201 in treating virus infections, even when lower doses and lower treatment volumes more akin to treating humans are used. DEF201 may serve as a means of quickly providing significant protection to first responders and medical chain personnel confronted with a deliberate release of variola or monkeypox virus into the environment, not to mention treating a variety of unrelated virus infections, many of which are important to both biodefense and public health.
